# Comparison of human adipose stromal vascular fraction and adipose-derived mesenchymal stem cells for the attenuation of acute renal ischemia/reperfusion injury

**DOI:** 10.1038/srep44058

**Published:** 2017-03-09

**Authors:** Liuhua Zhou, Qun Song, Jiangwei Shen, Luwei Xu, Zheng Xu, Ran Wu, Yuzheng Ge, Jiageng Zhu, Jianping Wu, Quanliang Dou, Ruipeng Jia

**Affiliations:** 1 Department of Urology, Nanjing First Hospital, Nanjing Medical University, No. 68 Changle Road, Nanjing, Jiangsu 210006, China; 2 Center for Renal Transplantation, Nanjing First Hospital, Nanjing Medical University, No. 68 Changle Road, Nanjing, Jiangsu 210006, China

## Abstract

Stem cells therapy has been suggested as a promising option for the treatment of acute kidney injury (AKI). This study was performed to compare the abilities of xenogenic transplantation of human adipose stromal vascular fraction (SVF) and adipose-derived mesenchymal stem cells (AdMSCs) to facilitate the recovery of renal function and structure in a rat model of ischemia/reperfusion (IR) induced AKI. SVF or AdMSCs were transplanted to the injured kidney through intra-parenchymal injection. Significantly improved renal function and reduced tubular injury were observed in SVF and AdMSCs groups. Administration of SVF or AdMSCs contributed to significantly improved cell proliferation and markedly reduced cell apoptosis in parallel with reduced microvascular rarefaction in injured kidney. IR injury resulted in higher levels of inflammatory cytokines, whereas xenogenic transplantation of SVF or AdMSCs reduced but not induced inflammatory cytokines expression. Additionally, *in vitro* study showed that administration of SVF or AdMSCs could also significantly promote the proliferation and survival of renal tubular epithelial cells underwent hypoxia/reoxygenation injury through secreting various growth factors. However, cell proliferation was significantly promoted in SVF group than in AdMSCs group. In conclusion, our study demonstrated that administration of SVF or AdMSCs was equally effective in attenuating acute renal IR injury.

Acute kidney injury (AKI), which is a common clinical syndrome, is considered to be an increasing global concern due to the increased patient morbidity and mortality, as well as the high risk for subsequent development of chronic kidney disease (CKD), despite the current advances in medical treatment^1^,^2^,^3^. Ischemia/reperfusion (IR) induced injury is a leading cause of AKI, which is associated with acute tubular-epithelial damage, loss of peritubular capillary, and inflammation^4^. Since the currently main therapies for AKI, including dialysis and renal transplantation, are often limited by the high expense and sever shortage of donor organs^5^, a new and effective method is in urgent need for the repair of AKI.

Recently, stem cell transplantation has become new candidate for the treatment of AKI. Simultaneously, adipose tissue has gained considerable attention as a cell source for tissue regeneration^6^. Studies have shown that adipose derived mesenchymal stem cells (AdMSCs) could effectively rescue IR induced kidney injury due to various mechanisms^7^,^8^,^9^,^10^. However, during the *in vitro* culture of AdMSCs to gain adequate cell numbers for *in vivo* administration, there are several concerns regarding the transplantation of cultured stem cells into human subjects, including the potential risks of xenogenic nutritional sources, microbial contamination, and so on^11^. In addition, the possible loss of the optimal opportunity for the repair of AKI is also a non-negligible issue due to the long-time cell culture *in vitro*. Fortunately, recent studies have suggested that uncultured adipose stromal vascular fraction (SVF) might be an attractive cell source for cell-based therapy on account of the real time isolation in an adequate quantity without *in vitro* expansion, which could avoid the risks described above^11^. In previous studies, both our team and other groups reported that autologous uncultured SVF could protect kidney from both IR and cisplatin induced acute injury^12^,^13^,^14^.

Despite the superiority of SVF compared to AdMSCs described above, no systematic study has compared the application of such two different cell types derived from adipose tissue for the treatment of AKI, as well as the efficacy and safety of them after being transplanted *in vivo*^12^,^13^,^14^. Moreover, though adipose tissue can be obtained through a minimally invasive method, the harvest of adipose tissue from a patient with serious illness, such as AKI, is still an invasive procedure, which may limit the application of autologous SVF therapy in the clinic. Due to the immunosuppressive property of AdMSCs, allogeneic or xenogeneic transplantation of AdMSCs into immunocompetent recipients is feasible and effective for the improvement of targeted diseases^15^. In addition, a recent study has demonstrated that xenogenic transplantation of human SVF successfully improved erectile function in diabetic mice without inducing systemic inflammation^16^. Therefore, to test the feasibility of xenogenic transplantation of human SVF to treat AKI, this study was performed to investigate the effects of xenogenic transplantation of uncultured human SVF on the repair of IR induced AKI in a rat model. Furthermore, we also compared the efficacy of human SVF and AdMSCs on the recovery of renal function and structure after AKI.

## Results

### Cell characterization

**Figure 1:**
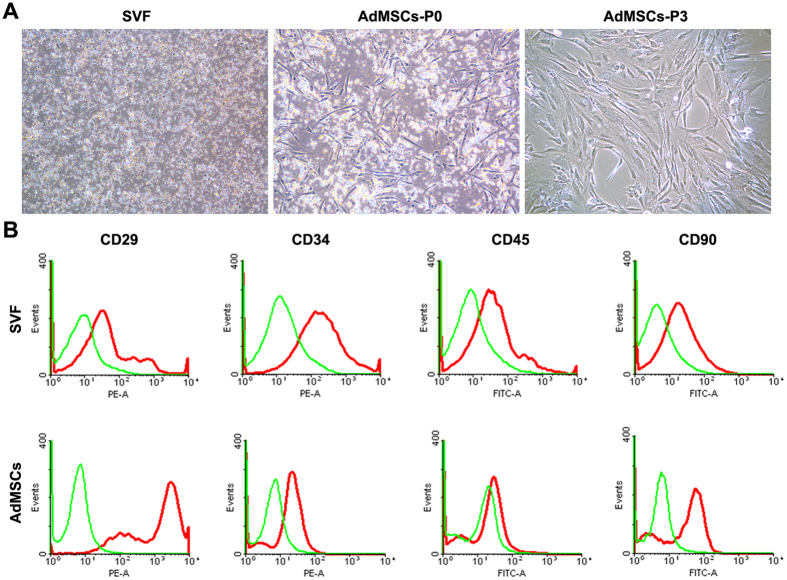
Characterization of SVF and AdMSCs. (**A**) Cell morphologies of freshly isolated SVF and cultured AdMSCs at passge 0 and 3. (**B**) Flow cytometric analysis of freshly isolated SVF and cultured AdMSCs at passge 3. Scale bars = 100 mm

SVF cells were successfully isolated from perinephric adipose tissue. After two days of culture, AdMSCs emerged with spindle-like morphologies. Cells were passaged when they reached over 80% confluence after about one week of culture. At passage 3, AdMSCs became fusiform and exhibited typical spindle shaped morphologies (Fig. 1A). Flow cytometry was performed to characterized SVF and AdMSCs by determining CD markers expression. Flow cytometric analysis demonstrated that SVF was positive for CD29 (29.7%), CD34 (7.9%), CD45 (15.8%) and CD90 (25.1%). AdMSCs were positive for CD29(99.8%), CD90(92.8%) and CD34(11.2%), but negative for CD45 (1.4%) (Fig. 1B).

### Effects of SVF and AdMSCs on renal tubular epithelial cells *in vitro* cultured in hypoxic condition

**Figure 2:**
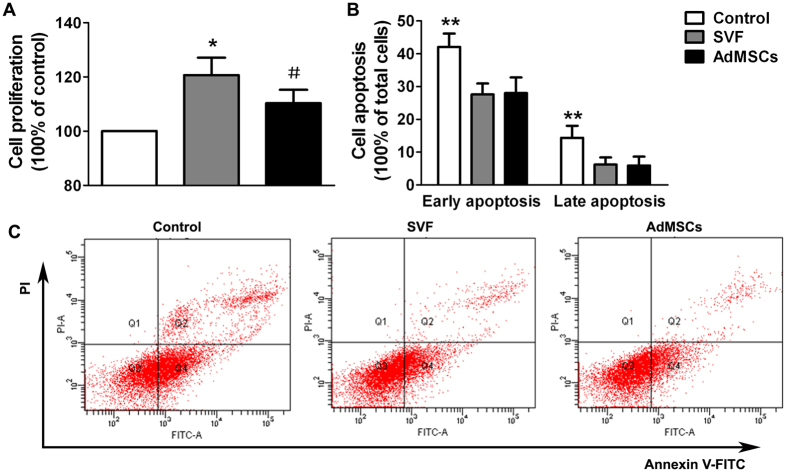
*In vitro* effects of SVF or AdMSCs on renal tubular epithelial cells in hypoxic environment. (**A**) SVF or AdMSCs administration could significantly promote the proliferation of hypoxic HK-2 cells, compared with control group. *p < 0.05, vs. Control and AdMSCs; ^#^*p* < 0.05, vs. Control. (**B**) SVF or AdMSCs administration could significantly reduce the apoptosis of hypoxic HK-2 cells, compared with control group. **p < 0.05, vs. SVF and AdMSCs. (**C**) Representative flow cytometry histograms of Annexin V/PI apoptosis assay in different groups.

Cell proliferation assay showed that both SVF and AdMSCs could significantly enhance the proliferation activity of HK-2 cells underwent H/R injury. In addition, the proliferation of HK-2 cells was significantly promoted in SVF group than that in AdMSCs group (Fig. 2A). Annexin V/propidiumiodide (PI) apoptosis assay was performed to assess H/R injury induced cell apoptosis of HK-2 cells, including early apoptotic cells (Annexin V positive, PI negative) and late apoptotic cells (Annexin V positive, PI positive). Results showed that both early and late apoptotic cells were increased after H/R injury. However, SVF or AdMSCs treatment could significantly reduce the proportion of apoptotic cells. Furthermore, no significant difference on the proportion of apoptotic cells could be found between SVF and AdMSCs groups (Fig. 2B,C).

ELISA showed that culture medium from SVF and AdMSCs contained significantly higher hepatocyte growth factor (HGF), vascular endothelial growth factor (VEGF) and stromal cell derived factor-1α (SDF-1α) than control medium, which indicated that both SVF and AdMSCs could secret HGF, VEGF and SDF-1α. However, HGF secretion from SVF was significantly higher than from AdMSCs (supplemental Fig. 1).

### Effects of SVF and AdMSCs on renal function and tubular injury

**Figure 3:**
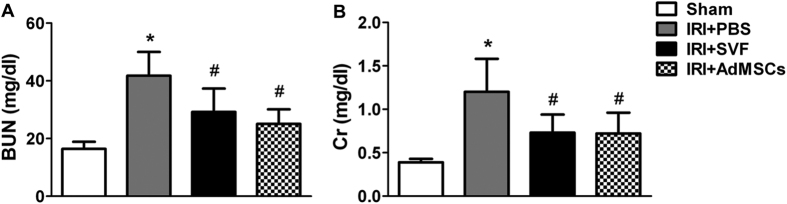
*In vivo* protective effect of SVF or AdMSCs on renal function in acute IR injuried kidney. Rats received SVF or AdMSCs administration showed significantly lower BUN (**A**) and SCr (**B**) values at 72 h after IR compared with control ones. There were no significant differences on the level of BUN and SCr between the SVF and AdMSCs groups. *p < 0.05, vs. Control, IRI + SVF and IRI + AdMSCs; ^#^*p* < 0.05, vs. Control.

**Figure 4:**
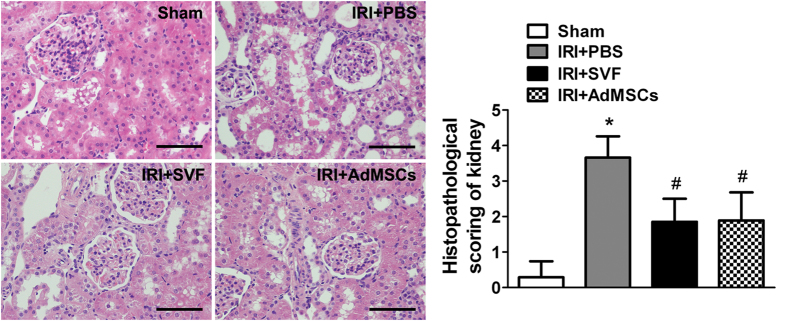
*In vivo* protective effect of SVF or AdMSCs administration on renal morphology in acute IR injuried kidney. Administration of SVF or AdMSCs contributed to reduced tubular injury compared with control group. There were no significant differences on tubular injury between the SVF and AdMSCs groups. *p < 0.05, vs. Control, IRI + SVF and IRI + AdMSCs; ^#^*p* < 0.05, vs. Control. Scale bars = 100 mm.

We compared the effects of AdMSCs and SVF on renal function and tubular injury in renal IR injury model. Animals in the control group showed a significant rise in serum levels of SCr and BUN compared with the sham group. However, a significant decrease in SCr and BUN was detected in both AdMSCs and SVF groups. Furthermore, there was no significant difference in renal function between the SVF and AdMSCs groups (Fig. 3). Histological examination showed that animals in control group subjected marked tubular injury, including cast formation, vacuolization, tubular necrosis, and so on, compared to sham group. SVF and AdMSCs treatment contributed to a significant reduction of tubular injury after IR injury. Furthermore, there were no significant differences in tubular injury between the SVF and AdMSCs groups (Fig. 4).

### Effects of SVF and AdMSCs on cell proliferation and apoptosis in injured kidney

**Figure 5:**
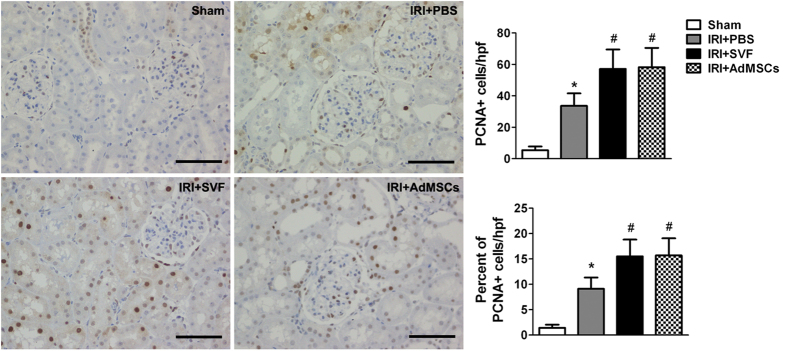
Immunohistochemical staining of PCNA in the kidney after acute renal IR injury. Administration of SVF or AdMSCs contributed to enhanced cell proliferation in injured kidney compared with control group. There were no significant differences on cell proliferation between the SVF and AdMSCs groups. *p < 0.05, vs. Control, IRI + SVF and IRI + AdMSCs; ^#^*p* < 0.05, vs. Control. Scale bars = 100 mm.

**Figure 6:**
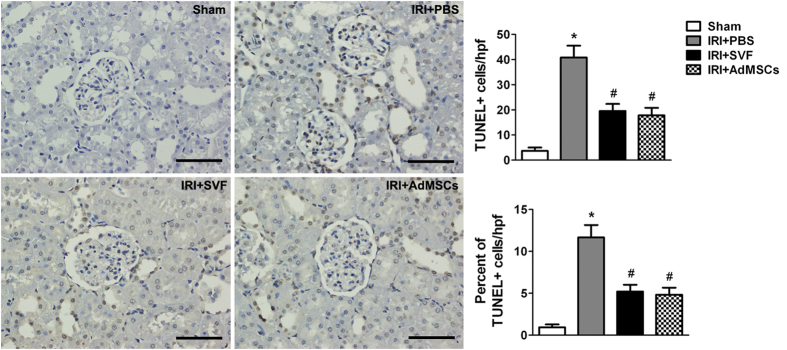
TUNEL staining of the kidney after acute renal IR injury. Administration of SVF or AdMSCs contributed to reduced cell apoptosis in injured kidney compared with control group. There were no significant differences on cell apoptosis between the SVF and AdMSCs groups. *p < 0.05, vs. Control, IRI + SVF and IRI + AdMSCs; ^#^*p* < 0.05, vs. Control. Scale bars = 100 mm.

The effects of SVF and AdMSCs on tubular cell proliferation were observed by evaluating PCNA expression in the kidney sections after IR injury. The enhancement of proliferation activity of renal tubular cells was demonstrated by the detection of increased number of PCNA positive cells in kidney sections. The number and proportion of PCNA positive cells was significantly increased after the treatment of SVF or AdMSCs. However, there was no significant difference on cell proliferation between the SVF and AdMSCs groups (Fig. 5). Additionally, the effect of SVF and AdMSCs on cell apoptosis was observed by evaluating TUNEL-positive cells in the kidney sections after IR injury. The number and proportion of TUNEL-positive cells were significantly increased in animals suffered from renal IR injury. SVF or AdMSCs administration could significantly reduce the number and proportion of TUNEL-positive cells. However, no significant difference on cell apoptosis was found between SVF and AdMSCs groups (Fig. 6).

### Effects of SVF and AdMSCs on microvasculature in injured kidney

**Figure 7:**
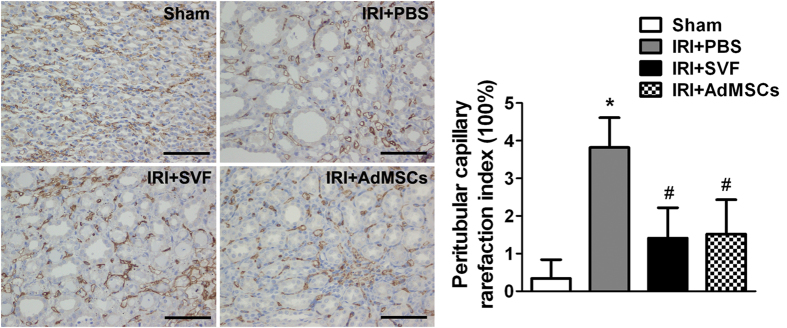
Immunohistochemical staining of CD34 in the kidney after acute renal IR injury. Administration of SVF or AdMSCs contributed to lower PCRI in injured kidney compared with control group. There were no significant differences on CD34 expression between the SVF and AdMSCs groups. *p < 0.05, vs. Control, IRI + SVF and IRI + AdMSCs; ^#^*p* < 0.05, vs. Control. Scale bars = 100 mm.

To evaluate the effects of SVF and AdMSCs on microvasculature, we detected the CD34 expression in the kidney sections after IR injury. PCRI was analyzed by detecting CD34 positive cells for the evaluation of peritubular capillary densities. The reduction of peritubular capillary densities was observed in animals suffered from IR injury. Treatment of SVF or AdMSCs could significantly increase the densities in injured kidney. The PCRI in SVF and AdMSCs groups was 1.41 ± 0.81% and 1.52 ± 0.91%, respectively, which were significantly lower than 3.82 ± 0.79% in control group (Fig. 7).

### Effects of SVF and AdMSCs on the expression of TNF-α and IL-10

**Figure 8:**
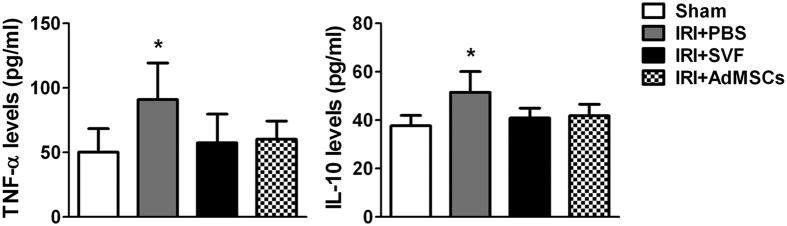
Expression of TNF-α and IL-10 in the serum of animals after acute renal IR injury. SVF or AdMSCs treatment contributed to lower expression of TNF-α and IL-10 in the serum of rats compared with control group. There were no significant differences on the expression of TNF-α and IL-10 between the SVF and AdMSCs groups. *p < 0.05, vs. Control, IRI + SVF and IRI + AdMSCs.

Serum levels of inflammatory cytokines, such as tumor necrosis factor-α (TNF-α) and interleukin-10 (IL-10), were detected by ELISA to evaluate the effect of xenogenic transplantation of SVF or AdMSCs on systemic inflammatory response. The levels of TNF-α and IL-10 were significantly increased in the rats underwent IR injury. After the treatment of SVF or AdMSCs, the levels of TNF-α and IL-10 were significantly reduced, while no significant difference could be found in both groups. (Fig. 8)

## Discussion

Stem cells therapy has been suggested as a promising option to repair AKI. SVF and AdMSCs are two different types of adipose tissue-derived cells, and both of them are able to facilitate tissue repair and regeneration via a variety of mechanisms^17^. However, compare with AdMSCs, SVF can be real time obtained in a sufficient quantity without *in vitro* culture, which can avoid the potential risk of microbial contamination during culturing procedures^11^. Therefore, uncultured SVF has been considered as more attractive cell source than cultured AdMSCs for tissue repair and regeneration, especially for the repair of acute organ injury including AKI. Despite both AdMSCs and SVF have been reported to be able to protect the kidney from IR injury^7^,^8^,^9^,^12^, no study has compared the effects of them on AKI. The present study was conducted to compare the therapeutic efficacy of intra-parenchymal injection of SVF or AdMSCs on renal IR injury. Additionally, the possible mechanism of their effects on AKI was investigated through an *in vitro* co-culture system that was applied to investigate the effects of SVF or AdMSCs on H/R induced injury of renal tubular cell.

Similar to a previous study, which compared SVF and adipose derived stem cells treatments in a rat model of cavernous nerve injury and showed that both cell types are equally effective in recovering penile erection^18^, the present study demonstrated that transplantation of SVF or AdMSCs also showed equally effective attenuation of acute renal IR injury. Both SVF and AdMSCs could improve renal function and structure after IR injury through enhancing tubular cell proliferation, increasing peritubular capillary densities, and inhibiting cell apoptosis, by acting in a paracrine way. We have also demonstrated that SVF or AdMSCs could secret various growth factors, including HGF, VEGF and SDF-1α, which might be responsible for the paracrine mechanism of SVF or AdMSCs attenuating renal IR injury. Yasuda *et al*.^13^ reported that subcapsular injection of SVF protected the kidney from cisplatin-induced acute injury by secreting renoprotective molecules, such as HGF and VEGF. SDF-1α is considered to be a regulator of angiogenesis and plays an important role in kidney repair^19^,^20^. In the present study, SVF or AdMSCs influenced the activity of renal tubular cell by secreting those growth factors transferred through a co-culture system. SVF showed stronger enhancement of renal tubular cell proliferation to AdMSCs *in vitro* but not *in vivo*, which might be attributed to the more secretion of HGF by SVF, because HGF is able to enhance the proliferation activity of renal tubular cell^21^.

Previously, studies mainly focused on the effect of cultured stem cells including AdMSCs on the repair of AKI, and proposed that they could facilitate renal repair through direct or indirect approaches. Li *et al*.^9^ reported that human AdMSCs promoted kidney tissue repair via direct differentiating into renal tubular epithelial-like cells. However, numerous studies have shown that the renoprotective effect of AdMSCs was attributable to their paracrine actions on the injured kidney by releasing soluble factors^21^,^22^,^23^. The application of uncultured SVF for the repair of kidney injury was firstly reported by Feng *et al*., who named it as uncultured adipose tissue-derived stem and regenerative cells, in a rat model of renal IR injury^12^. Subsequently, Yasuda *et al*.^13^ reported that SVF could ameliorate rat AKI induced by cisplatin through paracrine mechanism. In previous study, we had concluded that both pre-ischemic and post-ischemic administration of SVF could protect the kidney from IR induced injury via paracrine effect^14^. However, it remains unclear whether SVF cells could differentiate into renal tubular cells when transplanted *in vivo*. As is well known, SVF is a heterogeneous cell population in adipose tissue and contains plenty of stem/progenitor cells, including AdMSCs, endothelial progenitor cells (EPCs), and so on^24^. A previous study had shown that transplantation of SVF contributed to the regeneration of ischemic tissue in a rat model of chronic myocardial infarction through both differentiation and paracrine activity^25^. The direct differentiation of SVF cells into renal tubular cells may also play an important role in kidney tissue repair. Further study is needed to investigate whether direct differentiation activity of SVF taking part in kidney tissue regeneration by using cell tracking technique.

It has been confirmed that AdMSCs had immunosuppressive property due to the lack of major histocompatibility complex-II expression, which may be mediated by prostaglandin E2^15^,^26^. Studies have shown that xenotransplantation of human AdMSCs into different animal models was safe and effective for tissue repair and regeneration^15^,^27^,^28^. In a rat model of cisplatin-induced kidney injury, xenotransplantation of human AdMSCs led to decreased expression level of proinflammatory mediators^22^. Because SVF is a rich source of AdMSCs, it may also possess immunosuppressive property. Das *et al*.^16^ reported that xenotransplantation of human SVF into a diabetic mice model of erectile function did not induce systemic inflammation. In this study, decreased serum level of proinflammatory cytokine was detected after xenotransplantation of human SVF in a rat model of renal IR injury, which demonstrated immunosuppressive property of SVF. However, we have also found that xenotransplantation of human SVF or AdMSCs contributed to decreased serum level of IL-10, an anti-inflammatory cytokine. To further investigate immunosuppressive property of SVF, future study is needed to measure other anti-inflammatory cytokines, such as transforming growth factor-β1, which was reported to downregulate the secretion of IL-10 *in vitro*^29^. In addition, it is also needed to evaluate the possible rejection after xenotransplantation of human SVF or AdMSCs. Although we found decreased expression of TNF-α, which was reported to play an important role in allograft rejection^30^, more rejection markers should be examined in the future study.

One limitation of this study is that long-term effect of SVF and AdMSCs treatment on renal IR injury is not investigated, because even small change in kidney function can lead to both short-term and long-term complications^2^. Secondly, since SVF is a heterogeneous cell population and contains numerous cell types, the respective contributions of these cell types to the protective effect on renal IR injury should be evaluated in the future. At last, even though xenogenic transplantation of SVF or AdMSCs did not induce systemic inflammation, the systemic safety of xenogenic transplantation of the two cell types needs further investigated.

In conclusion, this study has confirmed that administration of SVF or AdMSCs contributed to the attenuation of IR induced tubular injury and improvement of renal function. Both SVF and AdMSCs are equally effective in improving renal function and structure after IR injury. However, SVF may be superior to AdMSCs when used for acute organ injury including AKI, because it can be obtained instantly in a sufficient quantity without *in vitro* expansion.

## Materials and Methods

### Cell isolation and culture

Perinephric adipose tissue was collected from eight patients (24 to 54 years of old) underwent abdominal operation and ruled out cancer, autoimmune diseases, etc. All the procedures involving humans were approved by the ethics committee of Nanjing First Hospital, Nanjing Medical University and informed consent was obtained from the patients. The procedures of SVF isolation were performed according to our previous protocol^31^. Briefly, the adipose tissue was rinsed with phosphate-buffered saline (PBS) and cut into small pieces, followed by incubation with 0.075% type I collagenase at 37 °C for 40 min in a shaking water bath. After being filtered with 200 μm nylon mesh and centrifuged at 400 g for 5 min to eliminate the mature adipocytes, the pellet was collected and treated with Red Blood Cell Lysis Buffer for 10 min, followed by being washed twice with ice-cold PBS. The nucleated cells was resuspended with PBS, counted with an automated cell counter, and then separated for the following experiments.

The SVF cells were plated in tissue culture flasks with Dulbecco’s modified Eagle’s medium (DMEM, Gibco) supplemented with 10% fetal bovine serum (FBS, Gibco) to *in vitro* expand AdMSCs. The culture flasks were maintained at 37 °C humidified atmosphere with 5% CO_2._ The medium was changed every 2 or 3 days until subconfluence for subculture.

### Flow cytometric analysis

Freshly isolated SVF cells and AdMSCs at passage 3 were collected, washed thrice with PBS containing 1% BSA, and then pelleted by centrifugation at 400 g for 5 min. Cell surface markers were examined by immunostaining with the following antibodies: phycoerythrin (PE) conjugated anti-CD29 (BioLegend), PE conjugated anti-CD34 (Bioss Inc), fluorescein isothiocyanate (FITC) conjugated anti-CD45 (BioLegend), and FITC conjugated anti-CD90 (BioLegend). The labeled cells were washed twice, resuspended and then analyzed with FACSCaliber (BD Biosciences). An isotype-matched IgG was set as the negative control for each primary antibody.

### Cell proliferation assay

Cell proliferation assay was performed as our previous protocol but with some modifications^14^. Briefly, HK-2 cells (1.5 × 10^3^/well) were seeded in the bottom chambers of HTS Transwell-96 system (Corning) and cultured in Thermo 3131 incubator (Thermo Fisher Scientific) for 24 h set at 37 °C, 1% O_2_, and 5% CO_2_. Then the plates were transferred into the normoxic humidified incubator at 37 °C with 5% CO_2_ and cultured for 2 h to induce hypoxia/reoxygenation (H/R) injury of HK-2 cells. Cells were divided into three groups: HK-2 cells cultured independently (control group), HK-2 cells co-cultured with SVF (SVF group), and HK-2 cells co-cultured with AdMSCs (AdMSCs group). SVF or AdMSCs (10^5^ cells for each) were seeded in the upper chambers. After 24 h of culture in normoxic condition, 10 μL of Cell Counting Kit-8 (CCK-8, Dojindo) was added into each well, and then the plates were incubated for 3 h. Finally, the absorbance of each well was measured at 450/620 nm on a microplate reader (Tecan).

### Annexin V/propidiumiodide apoptosis assay

HK-2 cells (50%–60% confluence) cultured in 24-well plates (Corning) were used for this assay. After H/R injury of HK-2 cells was induced, cells were divided into three groups as described above. The Milllicell^TM^ hanging Cell Culture Inserts (0.4 μm pore size, Millipore) were used for the co-culture of HK-2 and SVF cells (10^5^ cells for each). The plates were placed into the normoxic humidified incubator and cultured for 24 h. HK-2 cells were collected by using trypsin and stained with Annexin V Apoptosis Detection Kit FITC (eBioscience) according to the manufacturer’s instruction. At last, the proportion of apoptotic cells was measured by flow cytometry.

### Renal IR injury model and cell transplantation

Renal IR injury model was conducted according to previously described protocol but with some modifications^14^. A total of 32 male Sprague-Dawley rats (220 ~ 250 g) were used in this study. The animals were anesthetized by inducting with isoflurane and intraperitoneal administration of ketamine (100 mg/kg). The right kidney was removed by a midline abdominal incision. To establish the IR injury model, the left renal pedicle was blocked using a non-traumatic vascular clamp for 45 min, followed by removal of the clamp to initiate the left kidney reperfusion. PBS, SVF or AdMSCs were transplanted to the left kidney through intra-parenchymal injection. Animals were divided randomly into four groups: sham group (sham-operated); control group (injection with PBS); SVF group (injection with SVF); and AdMSCs group (injection with AdMSCs). 2 × 10^6^ SVF cells or AdMSCs suspended in 100 μL of PBS were used for cell transplantation. Three injections (poles and middle areas) were performed by using a 28-gauge needle.

Animals were housed in a standard room with constant temperature and humidity, on a 12 h light/dark cycle, and accessing to food and water *ad libitum*. All animal procedures were approved by the Institutional Animal Care and Use Committee of the Nanjing First Hospital, Nanjing Medical University. The investigation was performed in strict conformity with the institutional and national guidelines for laboratory animals.

### Renal function

Blood samples were collected for measurement of blood urea nitrogen (BUN) and serum creatinine (SCr) values at 72 h after IR injury in all four groups of rats (n = 8 for each group). Quantification of BUN and SCr concentrations was conducted by using standard laboratory equipment in our hospital.

### Histological analysis

The kidney specimens retrieved at 72 h after IR injury were fixed in 10% buffered formalin, gradually dehydrated, embedded in paraffin, cut into 5 μm sections, and stained with Hematoxylin and eosin (H&E). The degree of tubular injury was scored, based on HE staining in a blinded fashion, range from Grade 0 to 5 as previously described^32^. The scores reflected the severity of tubular damage as follows: 0, normal kidney; 1, ≤ 10%; 2, 11–25%; 3, 26–45%; 4, 46–75%; 5, ≥ 76%.

### Immunohistochemical analysis

Tissue sections (5 μm) were prepared as described above and used for immunohistochemical staining. After deparaffinizing and blocking, the sections were incubated at 4 °C overnight with rabbit anti-proliferating cell nuclear antigen (anti-PCNA) and rabbit anti-CD34 antibodies, respectively. Immunohistochemical assay for the staining of anti-PCNA and anti-CD34 was performed according to our previous protocol^33^. Peritubular capillary rarefaction index (PCRI) was analyzed by staining with anti-CD34 antibody and determined using the previously described procedures^19^,^34^. PCRI was determined by calculating the numbers of squares in 10 × 10 grids that was negative for CD34 staining. At least 10 nonoverlapping sequential fields at 400 x magnification were counted. The minimal PCRI score is 0, which indicates that no square in the grids is negative for CD34 staining, whereas the maximal score is 100, which means that all the squares are negative for CD34 staining.

### Enzymes linked immunosorbent assay

Levels of TNF-α and IL-10 in the serum of rats at 72 h after IR injury in the four groups were determined by Enzyme-linked immunosorbent assay (ELISA) kit (Uscn Life Science Inc) according to the manufacturer’s instruction. Levels of HGF, VEGF and SDF-1α in the culture medium of three groups after H/R in the section of Annexin V/propidiumiodide apoptosis assay were also determined by ELISA kit (RayBiotech). The absorbance was evaluated on a microplate reader (Tecan) and the concentration was calculated on the basis of standard curve.

### Statistical analysis

All data are expressed as mean ± SEM. One-way analysis of variance (ANOVA) was used to evaluate statistical comparisons of the data among groups. If ANOVA showed a significant difference, post hoc Tukey test was used to further evaluate the comparison between groups. Statistical significance was set at p < 0.05.

## Additional Information

**How to cite this article:** Zhou, L. *et al*. Comparison of human adipose stromal vascular fraction and adipose-derived mesenchymal stem cells for the attenuation of acute renal ischemia/reperfusion injury. *Sci. Rep.* **7**, 44058; doi: 10.1038/srep44058 (2017).

**Publisher's note:** Springer Nature remains neutral with regard to jurisdictional claims in published maps and institutional affiliations.

## Supplementary Material

Supplementary Figure 1
