# Supplementary material for: Comparison of human adipose stromal vascular fraction and adipose-derived mesenchymal stem cells for the attenuation of acute renal ischemia/reperfusion injury
Source: Sci Rep. 2017 Mar 9;7:44058. doi: 10.1038/srep44058 (PMC5343423; doi:10.1038/srep44058)
Supplement: Supplementary Figure 1 [file srep44058-s1.doc]

**Comparison of human adipose stromal vascular fraction and adipose-derived mesenchymal stem cells for the attenuation of acute renal ischemia/reperfusion injury**

Liuhua Zhou^1,2^, Qun Song^1^, Jiangwei Shen^1^, Luwei Xu^1,2^, Zheng Xu^1,2^, Ran Wu^1,2^, Yuzheng Ge^1,2^, Jiageng Zhu^1,2^, Jianping Wu^1,2^, Quanliang Dou^1,2^, Ruipeng Jia^1,2^

**Figure and Legend**


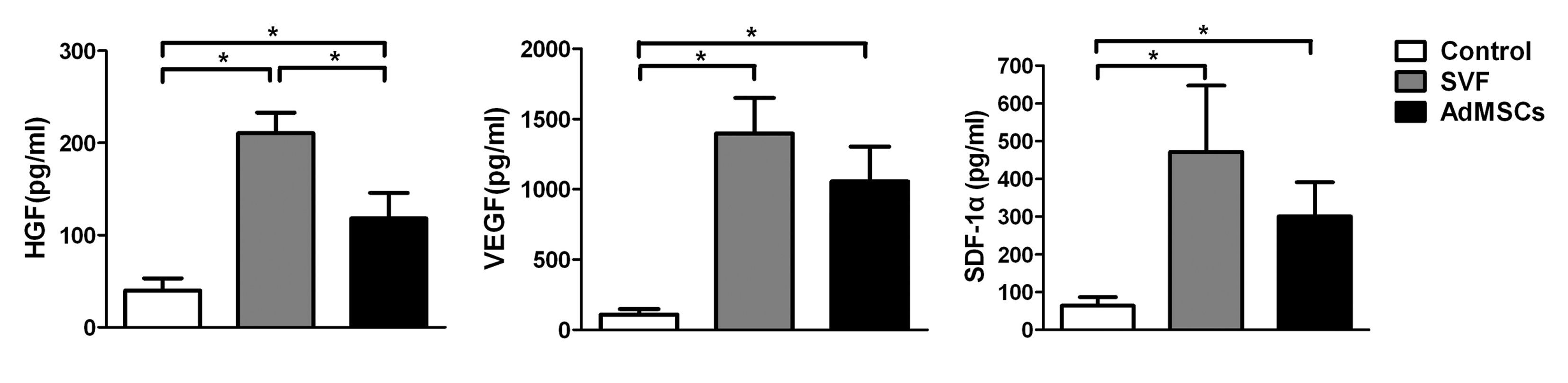


**Supplemental Fig.1.** **Secretion of hepatocyte growth factor (HGF), vascular endothelial growth factor (VEGF) and stromal cell derived factor-1α (SDF-1α) by SVF and AdMSCs**. ELISA showed that SVF and AdMSCs secreted significantly higher HGF (210.68±22.31 pg/ml for SVF, 118.13±27.81 pg/ml for AdMSCs, and 39.99±13.26 pg/ml for control), VEGF (1397.13±253.19 pg/ml for SVF, 1055.73±249.26 pg/ml for AdMSCs, and 108.62±41.26 pg/ml for control), and SDF-1α (471.77±175.49 pg/ml for SVF, 300.74±90.40 pg/ml for AdMSCs, and 63.63±23.23 pg/ml for control) than control medium. However, HGF secretion from SVF was significantly higher than from AdMSCs. **p*<0.05.
